# Enhancement of water diffusion and compression performance of crosslinked alginate films with a minuscule amount of graphene oxide

**DOI:** 10.1038/s41598-017-10260-x

**Published:** 2017-09-15

**Authors:** Ángel Serrano-Aroca, Juan-Francisco Ruiz-Pividal, Mar Llorens-Gámez

**Affiliations:** Bioengineering & Cellular Therapy Group, Facultad de Veterinaria y Ciencias Experimentales, Universidad Católica de Valencia “San Vicente Mártir”, C/Guillem de Castro 94, 46001 Valencia, Spain

## Abstract

A series of calcium alginate composite hydrogels with several calcium chloride contents ranging from 3 to 18 wt.% with and without 0.1 wt.% of graphene oxide (GO) was prepared in order to study the effect of crosslinking and nanofilling on water diffusion and compression performance. Thus, for high crosslinker contents, these composite hydrogels exhibited ultrafast diffusion of liquid water and excellent compression properties as compared with control (0 wt.% GO and the same crosslinking). These remarkable results are produced due to calcium cations are able to crosslink alginate and also graphene oxide nanosheets to form large crosslinked GO networks inside the calcium alginate hydrogels. Besides, these crosslinked GO/calcium alginate networks present nanochannels, as confirmed by electron microscopy, able to improve significantly water diffusion. Thus, these composite materials are very promising for many industrial applications demanding low-cost hydrogels with improved mechanical and water diffusion properties.

## Introduction

Hydrogels are very important hydrophilic materials due to their capacity of being able to absorb and retain large amounts of water within their structure^1^. Sodium alginate (SA) is a salt of alginic acid, a biopolymer of D-mannuronic (M) and L-guluronic acid (G) residues, with M and G present in different proportions and sequences, which depend on the alginic acid sources such as brown algae^2^ or microorganisms^3^. The gelation process occurs when divalent cations of Ca^2+^ interact with blocks of G residues to produce alginate gels with two contiguous, diaxially linked G residues forming a cavity that acts as a binding site for calcium atoms arranged according to the popular *egg-box model* buckled structure^4^. Calcium alginate is considered a very promising polysaccharide hydrogel for water treatment^5^, as an efficient alternative source for non-biodegradable plastic packaging materials^6^ and with many potential biotechnological applications^7,8^ since it is nontoxic, biodegradable, biocompatible and has a relatively low-cost in comparison with other polymeric materials. However, most hydrogels such as calcium alginate possess poor mechanical properties, such as the deformation behaviour under compression, because they are naturally brittle and cannot withstand the forces imposed, which restrict their widespread use in many industrial applications. Thus, alginates has been fabricated in various forms, such as films^9^, fibres^10^ and combined with other polymers^11,12^ using graphene oxide (GO) as reinforcement agent. GO shows excellent physical and chemical properties that are finding important applications in electronics, optics, energy, environmental science and biotechnology^13^ and its antibacterial activity^14^ render this kind of reinforcement even more promising for biomedical applications and other industries where contamination is a constant hazard to avoid. GO-based hydrogels has also many potential applications for molecular separation in desalination, wastewater treatment, water separation and purification due to the additional oxygen-containing functional groups present in GO and the increase in surface area^15–17^.

On the other hand, one of the most important current demands of the industries, which employ immobilised cells or enzymes in hydrogels (biocatalysts) such as calcium alginate^2^, is the improvement of mass transport to increase productivity by enhancement of water diffusion^8^. This improvement is also very desirable in many biomedical applications due to mass transport plays an important role in cell survival^18^.

In order to improve water diffusion in calcium alginate, GO was chosen as nanofiller due to the recent promising discoveries reported of fast diffusion of water nanodroplets on graphene being 2–3 orders of magnitude faster than the self-diffusion of water molecules in liquid water^19^, ultrafast permeation of liquid water through graphene-based nanochannels with a diffusion coefficient 4–5 orders of magnitude greater than in the bulk case^20^, ultrafast transport of water molecules in carbon nanomaterials such as membranes composed of an array of aligned carbon nanotubes^21,22^, and GO membranes^23,24^, with numerous hydrophobic graphitic nanochannels inside serving as pores. However, a very tiny weight percentage (0.1%) of GO was used with the aim of reducing the amount of nanofilling as much as possible because GO is still an expensive material. Therefore, it is very important to develop new nanocomposites minimizing production costs to render much more competitive any kind of future industrial product. As far as to our knowledge, no improvement of water diffusion and compression properties have been achieved before in calcium alginate hydrogels with such a small loading of GO (0.1 wt.%). Thus, in the present study, we hypothesize that the use this small amount of GO in the synthesis of calcium alginate composite hydrogels can affect differently depending on the amount of calcium chloride used in the reaction process. Water-soluble sodium alginate can form hydrogels in the presence of divalent cations of Ca^2+^ due to crosslinking via calcium bridges and the use of these these divalent cations have also been shown to be an effective way of interconnecting or producing crosslinking in carbon nanotubes^25^, and in GO^26^ through the bridging of their oxygen functional groups located on the basal planes and at the edges. Besides, our hypotheses also holds that for high crosslinker contents the water diffusion and compression properties of the composite hydrogels must be significantly improved (greater than in other hydrogels) as compared with control (0.0 wt.% GO with the same crosslinking density) because of the formation of large crosslinked GO networks, which create nanochannels in the composite hydrogel.

## Materials and Methods

### Materials

Sodium alginate was purchased from Panreac AppliChem (Germany) and was used as received. This sodium alginate was characterized by ^1^H-NMR and Gel Permeation Chromatography (GPC). Thus, the mannuronate/guluronate ratio was 1,56 and the average molar mass was 142.000 g/mol. Calcium chloride ($\ge$93.0%, Sigma-Aldrich) as crosslinker and graphene oxide powder (Sigma-Aldrich) were used without further purification.

### Synthesis

Alginate hydrogels with calcium chloride contents ranging from 3 to 18 wt.% (referred to the mass of SA) with and without 0.1 wt.% of GO were prepared using a procedure based on the synthetic method of direct mixing into alginate film-forming solutions with different amounts of calcium chloride^27^. Thus, 0.00025 grams of GO was mixed in 22 ml of distilled water under continuous stirring to get a homogeneous dispersion. After that, 0.25 grams of sodium alginate was added to this GO aqueous dispersion to be magnetically stirred for 1 hour at room temperature (24 ± 0.5 °C). The crosslinking solution was obtained by dissolving calcium chloride (3, 6, 12 or 18 wt.% with respect to the mass of SA) in 10 ml of distilled water. This solution was mixed thoroughly with the GO/SA aqueous solution under magnetic stirring and subsequently, the mixtures were cast onto transparent glass Petri dish and left undisturbed for 24 hours at 37 °C allowing formation of thin film. The films were peeled off from the mould and dry at 60 °C in vacuum to constant weight. The set of alginate films without GO were prepared following the same procedure but directly solving only 0.25 grams of sodium alginate in 22 ml of distilled water. These samples are hereafter referred to as A0, A3, A6, A12, A18 according to the wt.% of calcium chloride used in the synthesis and the composite hydrogels are named by adding the -GO suffix to the corresponding calcium alginate codes. These chemical routes were performed in triplicate to ensure reproducibility of the synthetic methods and physical characterisation.

### Characterisation

#### Liquid water sorption and diffusion

Liquid water sorption and diffusion was studied by means of immersion in liquid water to determine water uptakes as a function of time and calculate the apparent diffusion coefficients of water in the samples at 24 ± 0.5 °C from these measurements. This kind of experiments consisted in placing vacuum-dried samples at 60 °C in liquid water and weighing them at selected times after drying their surfaces with filter paper. These water sorption experiments were conducted in triplicate to confirm the reproducibility of the results. These low-cost and green synthetic procedures use distilled water as solvent and are conducted without consumption of thermal or sonic energy, very much in keeping with goals of green chemistry and sustainable technologies.

#### Electron Microscopy

Field Emission Scanning Electron Microscopy (FESEM, Zeiss Ultra 55 Model) with Energy-Disperse X-Ray Spectroscopy for elemental analysis was operated at an accelerating voltage of 2 kV to observe the morphology of GO at 100000x (elemental analysis at 20 kV) and A18-GO at a magnification of 30780x. High-Resolution Transmission Electron Microscopy (HR-TEM) of GO was employed using a JEM 2100 F JEOL 200 kV electron microscope dispersing the sample of GO with dichloromethane in an ultrasonic bath for ten minutes and subsequent drying at room temperature. Samples A6, A6-GO, A18 and A18-GO were examined in a scanning electron Jeol JSM-5410 microscope equipped with a cryounit Oxford CT 1500 using the low-temperature freeze drying technique (cryoSEM), which consisted firstly in immersing the samples in liquid nitrogen at low pressure (2·10^−2^ mbar) to freeze the open structure of the sponge. Afterwards, the samples were placed in the cryostage at the same pressure and at a temperature of −150 °C, where the samples were fractured and introduced in the sample stage at a higher vacuum (10^−5^ mbar) and at constant temperature of −85 °C for 30 min, which ensures the sublimation of water from the sample and prevents the porous structure to collapse. After that, the temperature is lowered again down to −150 °C to avoid structural changes and the samples are sputtered with a gold layer. Prior to examination the samples were swollen in liquid water for 2 minutes at 24 ± 0.5 °C in order to open the pores of the hydrogels. The micrographs of the swollen hydrogels were taken at an accelerating voltage of 20 kV in order to ensure a suitable image resolution.

#### Compression testing

Compression testing was performed using a TA-XT plus texture analyser (load cell 50 N) at room temperature (24 ± 0.5 °C). Stress versus strain measurements were plotted at a compression rate of 0.06 mm·s^−1^ from 0 to 100% strain. All samples were cut cylindrically using a 1 cm diameter custom designed punch for compression testing. These compression tests were conducted with at least six specimens. The thickness and diameter of each specimen after vacuum drying the samples at 60 °C for 48 hours were measured using a digital electronic calliper (ACHA, Spain).

## Results and Discussion

### Liquid water sorption and diffusion

**Figure 1:**
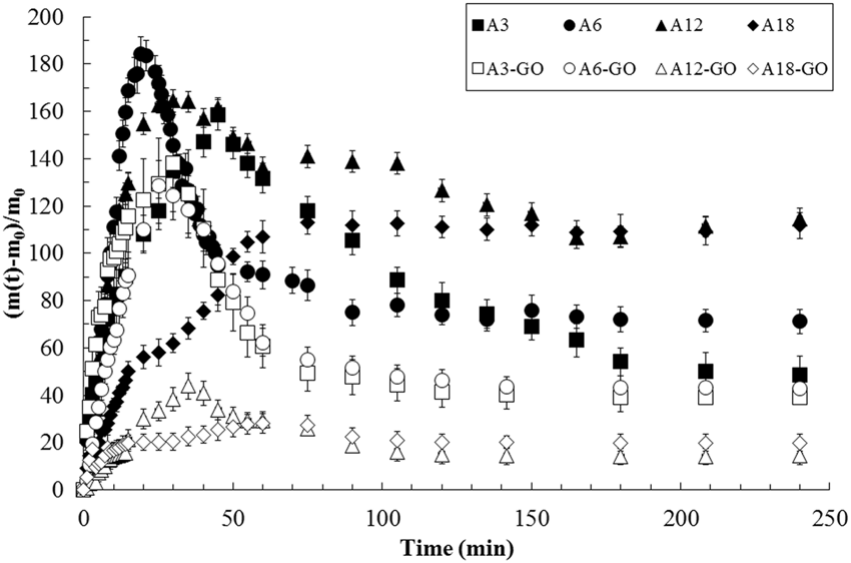
Water uptakes as a function of time for alginate with several crosslinking densities (solid symbols) and with 0.1 wt.% of GO (open symbols) at 24 ± 0.5 °C. Data are plotted as mean ± standard deviation.

Liquid water sorption was determined gravimetrically by measuring the weight of a sample as a function of time from the dry to the swollen state. Figure 1 shows a tremendous change in liquid water sorption when this tiny amount of GO is added to the calcium alginate hydrogels with different crosslinking densities. Besides, water sorption decreased dramatically with increasing the amount of crosslinker as expected, being this decrease more pronounced in the composites. The difference between the maximum and the last water uptake of the hydrogels decreased less and less with increasing crosslinker content as expected due to there are less uncrosslinked alginate chains available for dissolution during the water immersion experiment.

To significantly reduce water vapour sorption of sodium alginate is necessary the inclusion of at least 25 wt.% of graphene oxide sheets^28^. However, only 0.1 wt.% of GO is enough to produce a very significant decrease of liquid water absorption.

**Figure 2:**
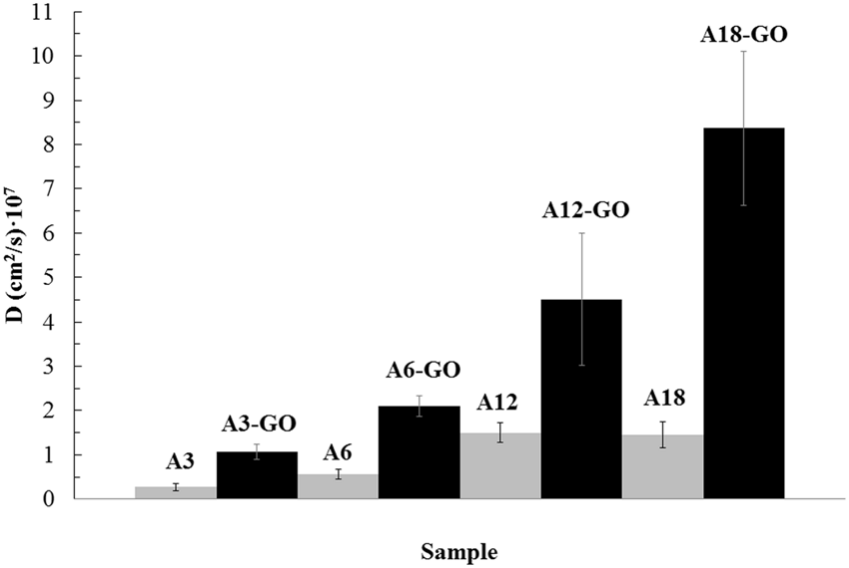
Apparent diffusion coefficients of liquid water (mean ± standard deviation) in calcium alginate hydrogels with different crosslinking densities with (black columns) and without (grey columns) 0.1 wt.% of GO.

Liquid water diffusion in these systems can be studied assuming that immersion in liquid water obey Fick’s law and Equation (1) holds for relatively small values of time *t*, corresponding to Δ*m*_*1,t*_/Δ*m*_*1,∞*_ < 0.5.

1 $$\frac{{\rm{\Delta }}{m}_{1,t}}{{\rm{\Delta }}{m}_{1,\infty }}\approx 4{(\frac{Dt}{\pi \cdot {l}^{2}})}^{\frac{1}{2}}$$

where *Δm*_*1,t*_ and *Δm*_*1,∞*_ are the weight gains at time *t* and at equilibrium, respectively, *l* is the sample thickness and *D* the diffusion coefficient. The maximum water contents of Fig. 1 were used to calculate *Δm*_*1,∞*_ in the representation of *Δm*_*1,t*_/*Δm*_*1,∞*_ vs. *t* ^*1/2*^ */l* showing that the water liquid diffusion mechanism of all liquid water sorption curves is non-Fickian^29^, which is in very good agreement with earlier studies performed with liquid water in alginate-based semi-interpenetrating polymer networks^30,31^, and with water vapour in alginate films^32,33^ and (reduced) graphene oxide-alginate composites^28^. However, an apparent diffusion coefficient can be determined with Equation (1) fitted in the linear part of all these plots for small values of time to be able to compare the liquid water diffusion capacity in all these materials. These results showed that a tiny weight percentage of this nanomaterial can produce a huge water diffusion improvement of up to almost 6 times in calcium alginate composite hydrogels (see A18-GO and A18 in Fig. 2). Crosslinking density can also increase the diffusion coefficient of alginate hydrogels. However, the increase produced per mass of calcium chloride added in comparison with that of GO is tremendously much lower.

**Figure 3:**
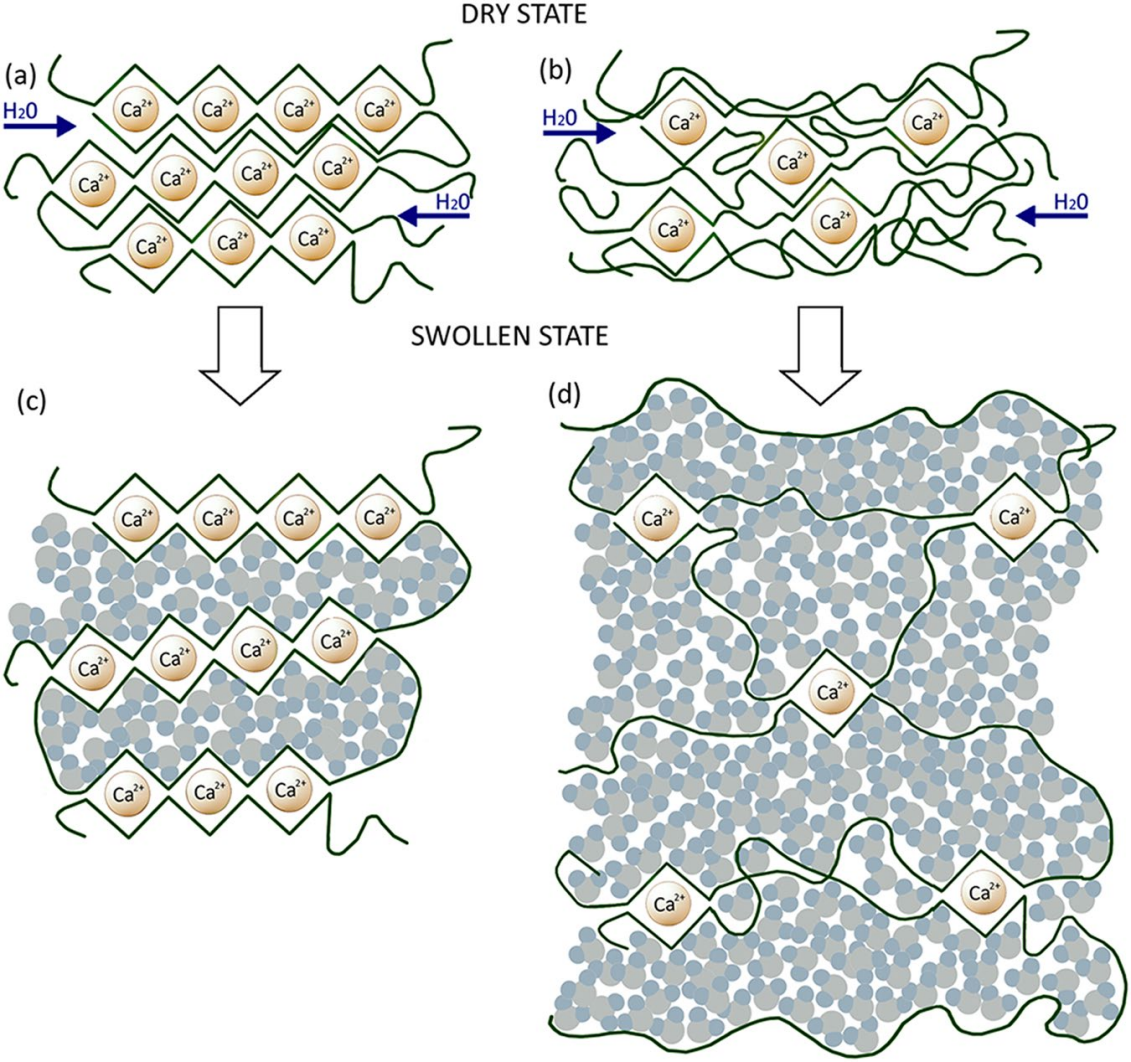
Dry and swollen state representation of calcium alginate hydrogels in liquid water for high (**a** and **c**) and low (**b** and **d**) crosslinker contents.

The diffusion coefficient of alginate probably increase with crosslinking density because these *egg-box* junctions present in the calcium alginate sample must create discontinuities in the dry state where water can diffuse faster (see Fig. 3(a)). However, the maximum capacity of swelling decreases with crosslinking density as expected (see Fig. 1). When the amount of crosslinker is too low as in A3 or A6, water needs to separate the polymer chains in order to penetrate rendering water diffusion slower but, on the contrary, the calcium alginate hydrogels are able to swell much more (see Fig. 3(b and d)) with some dissolution of the uncrosslinked polymer chains.

### Electron Microscopy

**Figure 4:**
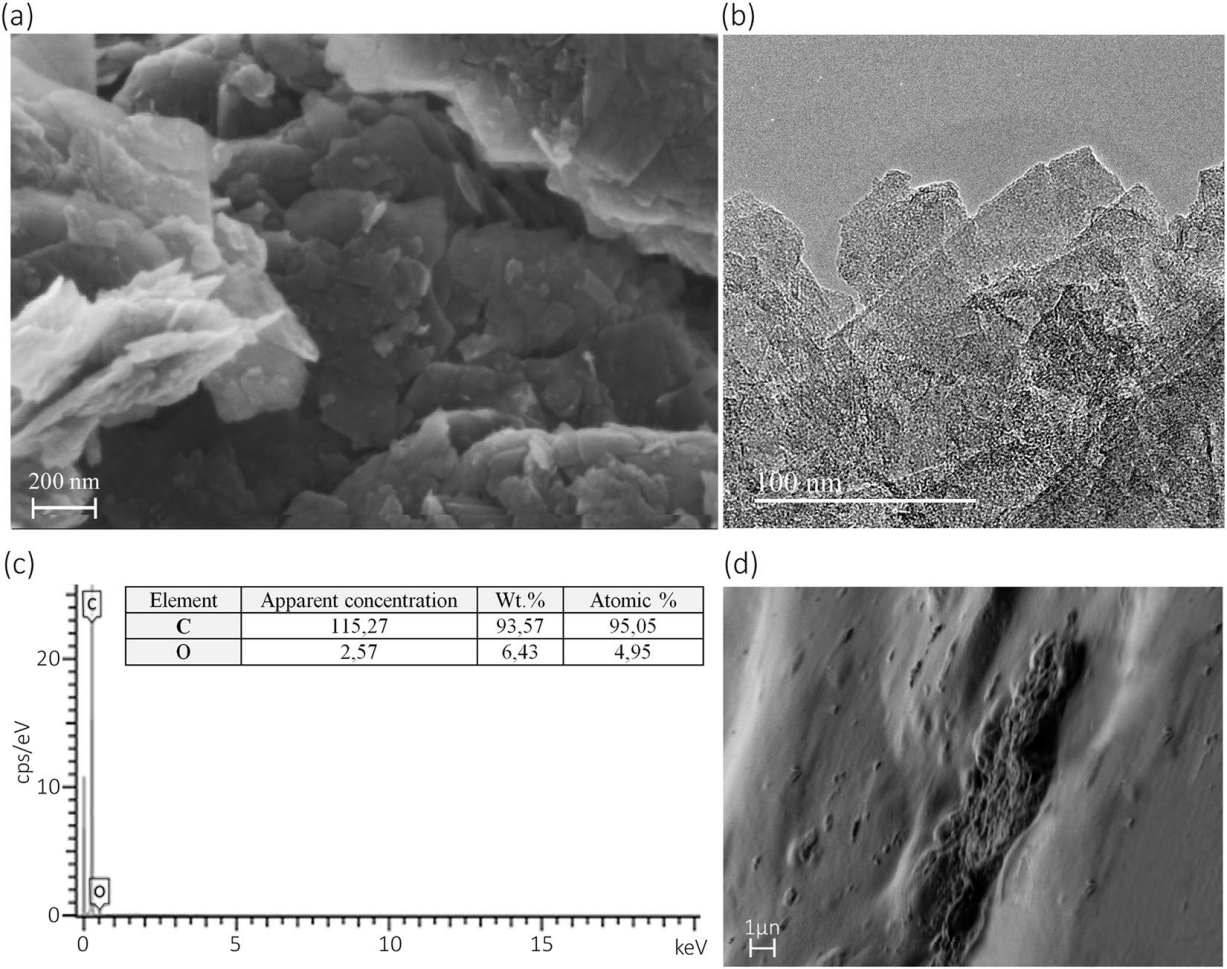
FESEM at 100000x (**a**), HR-TEM (**b**) and Elementary Analysis (**c**) of graphene oxide and FESEM of dry A18-GO (cryogenic fracture) at 30780x (**d**).

The GO powder used in this work present the morphology of nanosheets of approximately 100–200 nm length stacked together by van der Waals forces and π-π interactions (see Fig. 4(a and b)). The elementary analysis of GO (Fig. 4(c)) showed a composition of C and O elements in accordance with that given by the manufacturer.

**Figure 5:**
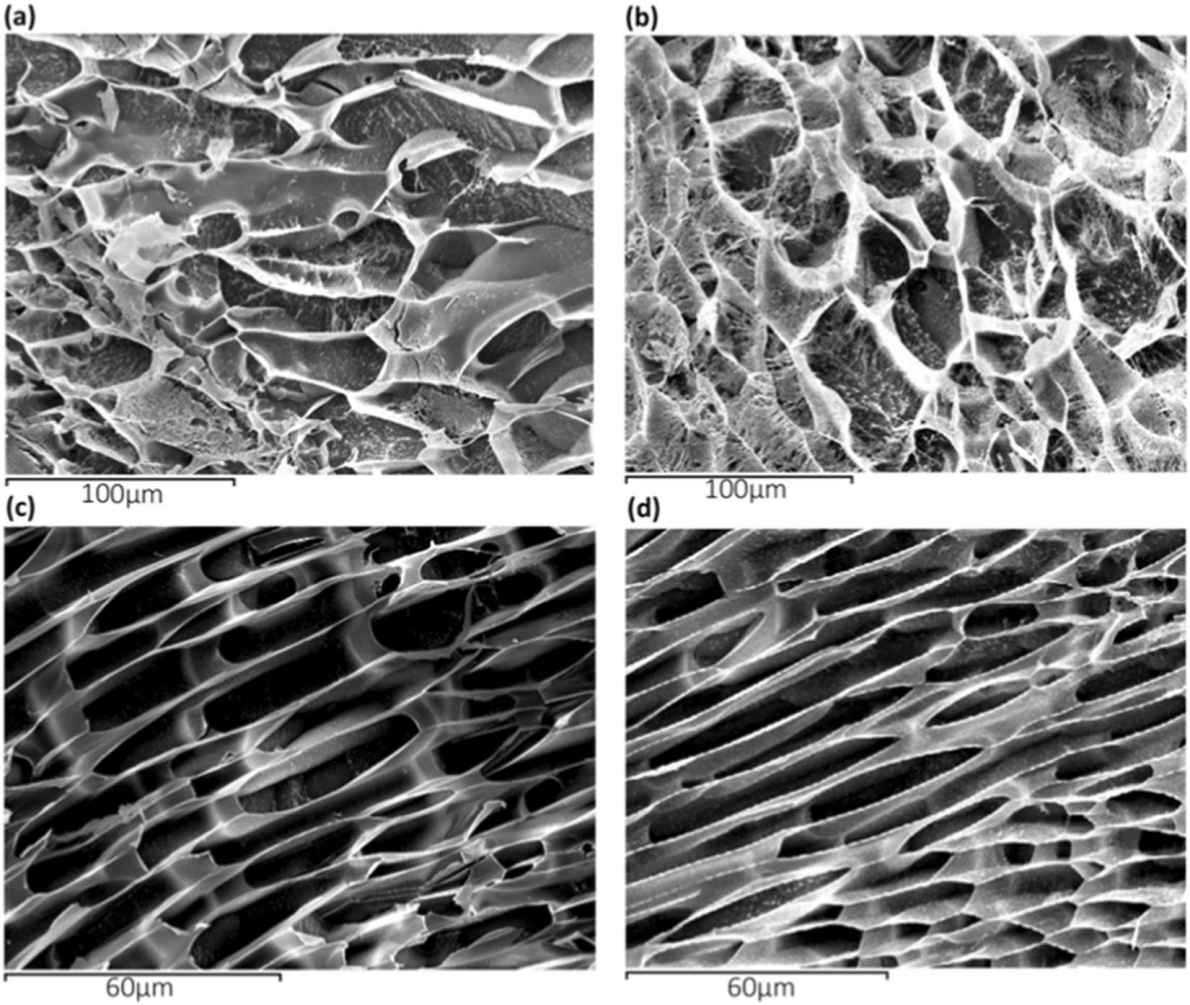
Effect of crosslinking and GO filling in the swollen state on the CryoSEM morphology of A6 (**a**), A6-GO (**b**), A18 (**c**) and A18-GO (**d**) at the same magnifications (500x and 1000x) after two minutes of immersion in water at 24 ± 0.5 °C.

In the dry state, the porosity of A18 collapse closing all pores observed after swelling in water (see Fig. 5(c)). However, the porosity of dry A18-GO is not totally collapsed showing nanochannels in some parts of the cryogenic fracture due to the presence of large crosslinked GO networks in the calcium alginate composite hydrogels (see Fig. 4(d)).

This nanoporosity created by these large crosslinked GO networks let liquid water to diffuse very fast through these nanochannels and explains somehow the high increase of water diffusion (Fig. 2) and the high decrease of water sorption in A18-GO (Fig. 1).

CryoSEM was performed in order to study the effect of crosslinking and GO filling on the porous morphology after 2 minutes of immersion in liquid water at room temperature (Fig. 5).

CryoSEM analysis showed that the morphology of A6 is much more heterogeneous than A18 (see Fig. 5(a and c)) with the alginate polymer chains disposed in the form represented schematically in Fig. 3(d) and (c) respectively. The morphology of these hydrogels are quite similar to those with GO for similar crosslinking densities (see Fig. 5(b and d)). However, for high calcium chloride contents, GO nanosheets react by coordination chemistry with the divalent cations of Ca to form crosslinked GO networks, which produce a composite hydrogel with more flattened elliptical pores.

### Compression testing

**Figure 6:**
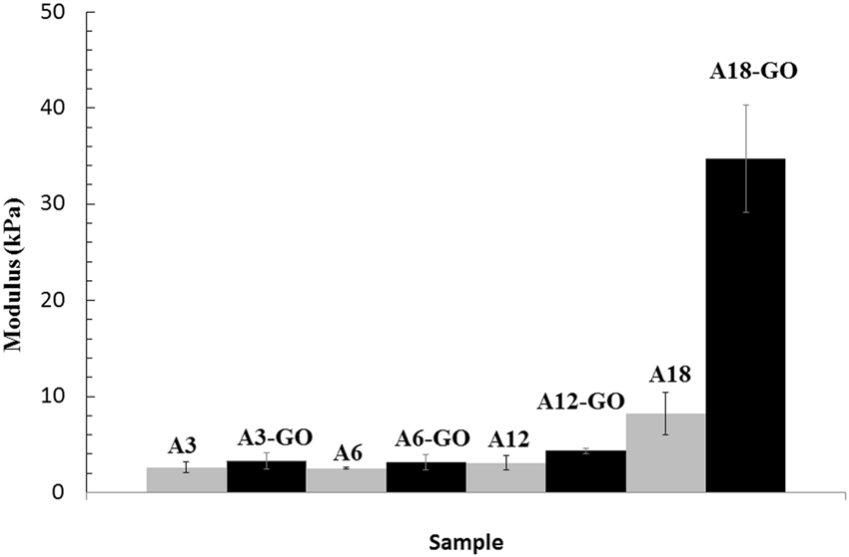
Compression properties of the dry hydrogels with different crosslinker contents plotted as mean ± standard deviation.

The compressive modulus of A3 and A6 slightly increased with the addition of 0.1 wt. of GO (see Fig. 6). However, this improvement of compression performance increased more and more with increasing crosslinker content. Thus, sample A18-GO showed a compressive modulus more than 4 times higher than that of A18. This enhancement of mechanical properties can also be slightly appreciated in the swollen structure of A18-GO with no sign of broken material (see Fig. 5(d)) as seen in A18 (Fig. 5(c)). This fact can also be explained by the complexation reaction between the GO nanosheets and the Ca^2+^ cations, which render calcium alginate hydrogels much more resistant because of having continuous crosslinked GO networks. Thus, the composite hydrogels such as A3-GO and A6-GO has not enough number of calcium atoms to form large GO networks able to improve compression so much. However, with increasing the amount of crosslinker content present in the reactive mixture, the GO networks are able to grow and become larger and larger increasing the compressive modulus more and more.

The measured compressive modulus for these alginates with different crosslinking densities compares well with previous studies reporting values of ~3 kPa^34^ for a calcium alginate gel (*Laminaria digitata*, M/G = 1.6, molecular weight of 620.000 g/mol) and 9.6 ± 2.0 kPa^35^ for another kind of calcium alginate (*Macrocystitis pyrifera*, M/G = 1.67, molecular weight of 50.000 g/mol). However, a compressive modulus of ~31 kPa^36^ was determined in a more recent study of a third type of calcium alginate (*Laminaria hyperborea*, M/G = 0.49, molecular weight of ~200.000 g/mol), which likely reflects that differing test protocols for measuring compressive stiffness and the fact that differences in alginate composition are likely to generate differences in material properties, specifically the M/G ratio. Thus, a calcium alginate with more G blocks can produced a very significant increase of the compressive modulus^37^. Anyway, the reinforcement produced by the addition of this small amount of GO in the alginate used in this study is evident, especially for high crosslinker contents. Besides, this mechanical improvement, which is able to crosslink both components (alginate and GO), is much more significant than those found in other hydrogels such as poly(vinyl alcohol) using similar wt.% of GO^38^ prepared from natural graphite through a modified Hummers method^39^ or employing boron-crosslinked GO^40^ in good agreement with our starting hypothesis.

## Conclusion

In conclusion, the effect of crosslinker content on water diffusion and compression was studied in GO/calcium alginate composite hydrogels showing that a very significant increase of water diffusion (almost 6 times faster) and mechanical compressive modulus (more than 4 times higher) can be achieved with higher crosslinker contents with respect to the corresponding reference samples of calcium alginate with the same crosslinking density. These findings support our hypothesis that the use of even a small amount of GO in the synthesis of these composites can improve the compression and water diffusion properties depending on the amount of calcium chloride used in the synthetic procedure due to large crosslinked GO networks can be formed by coordination chemistry with Ca^2+^ ions in the calcium alginate composite hydrogel producing nanochannels able to improve tremendously water diffusion and mechanical compression at the same time. These achievements render these hydrophilic materials very promising for many industrial applications such as heterogeneous catalysis, tissue engineering, odontology and biodegradable packaging materials with higher compression and liquid water diffusion requirements.
